# The future treatment for type 1 diabetes: Pig islet- or stem cell-derived β cells?

**DOI:** 10.3389/fendo.2022.1001041

**Published:** 2023-01-05

**Authors:** Raza Ali Naqvi, Afsar Raza Naqvi, Amar Singh, Medha Priyadarshini, Appakalai N. Balamurugan, Brian T. Layden

**Affiliations:** ^1^ Department of Periodontics, College of Dentistry, University of Illinois at Chicago, Chicago, IL, United States; ^2^ Department of Surgery, University of Minnesota, Minneapolis, MN, United States; ^3^ Department of Medicine, College of Medicine, University of Illinois at Chicago, Chicago, IL, United States; ^4^ Center for Clinical and Translational Research, Nationwide Children’s Hospital, Columbus, OH, United States; ^5^ Department of Pediatrics, College of Medicine, The Ohio State University, Columbus, OH, United States

**Keywords:** adult pig islets (API), neonatal pig islets (NPIs), maximum survival time (MST), gal knockout (GTKO) pigs, microencapsulation, instant blood-mediatedinflammatory reaction (IBMIR), stem cell derived beta cells

## Abstract

Replacement of β cells is only a curative approach for type 1 diabetes (T1D) patients to avoid the threat of iatrogenic hypoglycemia. In this pursuit, islet allotransplantation under Edmonton’s protocol emerged as a medical miracle to attain hypoglycemia-free insulin independence in T1D. Shortage of allo-islet donors and post-transplantation (post-tx) islet loss are still unmet hurdles for the widespread application of this therapeutic regimen. The long-term survival and effective insulin independence in preclinical studies have strongly suggested pig islets to cure overt hyperglycemia. Importantly, CRISPR-Cas9 technology is pursuing to develop “humanized” pig islets that could overcome the lifelong immunosuppression drug regimen. Lately, induced pluripotent stem cell (iPSC)-derived β cell approaches are also gaining momentum and may hold promise to yield a significant supply of insulin-producing cells. Theoretically, personalized β cells derived from a patient’s iPSCs is one exciting approach, but β cell-specific immunity in T1D recipients would still be a challenge. In this context, encapsulation studies on both pig islet as well as iPSC–β cells were found promising and rendered long-term survival in mice. Oxygen tension and blood vessel growth within the capsules are a few of the hurdles that need to be addressed. In conclusion, challenges associated with both procedures, xenotransplantation (of pig-derived islets) and stem cell transplantation, are required to be cautiously resolved before their clinical application.

## 1 Introduction

Type 1 diabetes (T1D) is a devastating disease, where glucose control in the body gets askew due to the destruction of β cells in the pancreas by auto-reactive CD8^+^ T cells (1, 2). In the United States alone, approximately 1.5 million people suffer from T1D (3). Therefore, for subjects with T1D, taking insulin is not an option but a lifesaving commodity. Though insulin therapy attempts to maintain normal glucose homeostasis in T1D patients, iatrogenic hypoglycemia due to impaired insulin–glucagon counter-regulatory responses (4) is a major shortcoming of this treatment. Importantly, hypoglycemic episodes can be fatal in T1D (5–7), and new treatment strategies, either alone or in conjunction with current modalities, aimed at both hyperglycemia and hypoglycemia management in T1D patients are utmost required.

In the year 2000, James Shapiro developed a protocol, dubbed as the Edmonton protocol, for the transplantation of allo-islets under steroid-free immunosuppressants. In this study, all patients had demonstrated repeated episodes of severe hypoglycemia before the islet transplantation. This procedure maintained normoglycemia in T1D patients without a threat of hypoglycemia and had dramatically improved their quality of life (8).Shortage of cadaveric pancreas donors challenged the widespread application of allo-islet transplantation (9). Owing to anatomical and physiological similarities, pig is now being considered an alternative option (10), and various groups achieved long-term survival of pig islets in cynomolgus monkeys for more than a hundred days (11, 12). Despite the great success of allotransplantation and then xenotransplantation of islets, 60% islet loss within 2 to 3 days in either case (via intraportal transplantation strategy) turned out to be one of the hurdles in restoring adequate islet mass secreting enough amount of insulin. Induction of instant blood-mediated inflammatory reaction (IBMIR) as a result of activation of thrombosis and complement pathways is the sole reason for post-transplantation islet loss (13–16). A multitude of strategies are now being attempted to reduce instant islet loss as a result of IBMIR (17–19). One recent advancement in this direction is CRISPR-Cas9-mediated genetic modification for the development of IBMIR-resistant pigs. Pig islets expressing human-derived complement-regulatory protein (CD46) and tissue factor (CD39), either alone or in combination, turned out to be beneficial in maintaining a state of normoglycemia in diabetic monkeys for more than 1 year. Lately, abrogation of IBMIR in xenotransplantation of neonatal pig islet cell cluster in nonhuman primates (NHPs) by GGTA^-/-^ CD55^+^CD59^+^ pigs further proved the hypothesis (20, 21).

**Figure 1:**
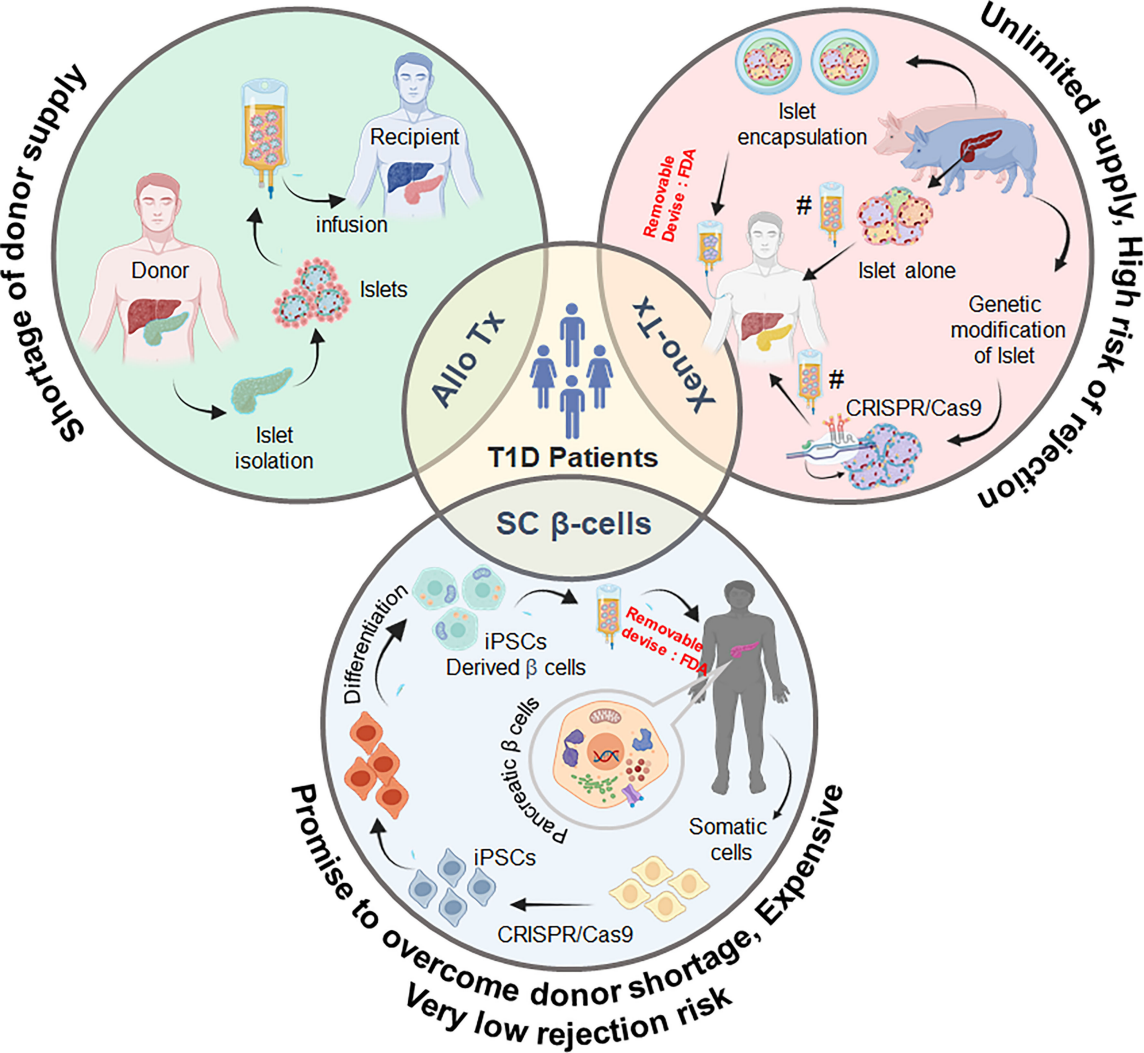
Schematic representation of the promising sources of pancreatic β cells and possible strategies for the treatment and management of type 1 diabetes. Tx, transplantation; SC, stem cell; iPSC, induced pluripotent stem cells. As per FDA recommendations, any xeno- or stem cell-derived cell will have to be contained in a removable device for use in humans.

In addition to IBMIR, immune incompatibility between donor and recipient is another major reason for xenogeneic rejection, and a potent immune suppression regimen is therefore needed to overcome xeno-immunity. Ironically, these permanent immune suppressants have shown to be deleterious and toxic for both islet grafts as well as recipients. Therefore, encapsulation of islets has been attempted in various clinical trials to rescue the pig islets from the recipient’s immune response (22). Though islets encapsulated in various kinds of biocompatible materials are now being used in clinical trials, the biocompatibility of the encapsulating material is still a matter of debate. The concept of SC-derived β cells has also proven fortuitously beneficial to minimize the use of immunosuppressive drugs and to increase the antigen similarity of the islets to be used for transplantation with the recipient. Along these lines, the development of induced pluripotent stem cell (iPSC)-generated β cells is also under progress. Two groups—Douglas Melton’s and TJ Kieffer’s—independently achieved a major success in the conversion of iPSCs into functional β cells (23, 24). Scaling up this process to obtain the total number of β cells required to restore normoglycemia (*i*.*e*., ~10^9^ cells/average weight of human: 70 kg) and to protect transplanted β cells from an aberrantly activated immune system in T1D patients are two hurdles to be resolved before transitioning them towards the clinic. In this review, we discuss the challenges associated with human and pig islet transplantation and explore whether stem cells could evolve as better technology to cure T1D (**Figure 1**).

## 2 Allotransplantation of human islets

Insulin therapy is dependent on the absorption and clearance rates of injected insulin (4). Importantly, T1D patients under intensive insulin therapy gradually develop the attenuation of sensitivity towards hypothalamus-dependent insulin-regulating glucagon–epinephrine responses (25, 26). To prevent loss of these glucose counter-regulatory responses, cell-based therapy—islet transplantation—may be best suited to prevent overt hyperglycemia in T1D patients (8). Overall, islet transplantation is less invasive than whole pancreas transplantation—the patient of which, most of the time, suffers from complications due to the drainage of exocrine secretion into the intestine and/or the bladder (27). Islet transplantation, pioneered by Paul Lacy in 1967, was first used to restore normoglycemia in rats (28). Subsequently, in the year 2000, James Shapiro, under the Edmonton protocol, successfully reported insulin independence in all of the seven recipients (T1D patients) at 1 year after transplantation. Steroid-free immune suppression, multiple-donor islet transplantation (more than one time), fine-tuning of isolation method, and immediate transplantation of islet without culture are the salient features of the Edmonton protocol (8, 29). The results of the Edmonton protocol were further substantiated by the Immune Tolerance Network multi-center trial (30). Furthermore, a more recent phase 3 trial of transplantation of human islets in T1D demonstrated the successful achievement of insulin independence without severe hypoglycemic episodes (31). In this study, the achievement of two imperative goals were interrogated after human islet transplantation (1): primary endpoint included the reduction of HbA1c level of <7.0% (53 mmol/mol) (2), the glycemic goal encompassed the independence from severe hypoglycemic episodes (SHEs) from day 28 to 365 after the first islet transplant. Interestingly, 87.5% of islet transplantation subjects achieved the first endpoint for 1 year, and 71% individuals had demonstrated it for 2 years. The median HbA1c level was maintained as 5.6% (38 mmol/mol) in both years. Furthermore, these individuals have highly significant improvements in Clarke and HYPO scores (*P* > 0.0001)—hypoglycemia awareness. Therefore, human islet transplantation fostered the protection from SHEs in subjects who had earlier shown significant episodes of intractable impaired awareness of hypoglycemia and SHEs even after insulin treatment (31). Importantly, the median insulin use was dramatically dropped from 0.49 to 0.13 units/kg at day 75 and 0.00 units/kg at day 365 (*P* < 0.0003) in these subjects. Individuals with functioning islet graft also demonstrated basal or stimulated serum C-peptide level as >0.3 ng/ml (31).

## 3 Xenotransplantation promises a relentless supply of good-quality islets

The successful application of islet transplantation under Edmonton protocol suffers from the shortage of adequate donor islets. Even if we consider one pancreas per T1D patient, there would still be an extreme shortage of donors to establish it as a widespread clinical reality. Importantly, sometimes two or more donors are needed to induce long-term normoglycemia in T1D patients due to the loss of islets in the first 60 min of intraportal transplantation *via* IBMIR (8). In this pursuit, the concept of xenotransplantation of pig islets promised to resolve the islet donor shortage (10, 29, 32–34).

Pig is considered as a good source of islets due to the following reasons (1): human and pig insulin differs only by one amino acid (35), as exemplified by the fact that humans have been treated successfully with porcine insulin for >40 years (10) (2); adult pig pancreas consists of an ample number of islets that respond to glucose stimulation (3); pigs are easily bred, sacrificed for food, and have large litters with offspring that rapidly attain adult size (4); pig islets, if coming from a single donor, could be more consistent as compared with human cadaveric islets that have different degrees of functionality, cold ischemia injury, and islet damage due to the presence of disease (5); the autoimmune responses against pig islets are different from those against human islets; and (6) genomic editing of pigs with required immune-modulatory gene(s) can make pig islets less prone to degradation mechanisms (10, 36). Despite all these advantages, the glucose-stimulated insulin secretion capacity of adult and juvenile pig islets was reported as one-third compared with human islets. Muller et al. performed glucose-stimulated insulin secretion (GSIS) on human islets, adult pig islets (APIs), and juvenile pig islets (JPI) by challenging them separately with basal glucose (2.5 mM) and high glucose (16.7 mM). This study demonstrated that GSIS of API and JPI was one-third and one-sixth, respectively, compared with human islets, thereby emphasizing that a substantially higher dose of porcine islets is needed to reverse hyperglycemia compared with human islets (37).

### 3.1 Neonatal and adult porcine islets restored long-term hyperglycemia

Initially, due to the easy isolation process, fetal pig islet-like cell clusters (ICCs) and neonatal pig islets (NPIs) have been used to reverse hyperglycemia in mice studies. In addition to their easy isolation process, ICCs and NPIs are apparently resistant to ischemic and inflammatory damage. Despite these advantages, the following demerits questioned their clinical applicability: 2 to 3 months of maturation time to achieve *in vivo* functionality (38, 39), poor insulin response to glucose (40, 41), and complete destruction of ICCs within 12 days post-transplantation in diabetic monkeys (42). After that, the transplantation biologist focused on neonatal pig islets—comprised of differentiated pancreatic endocrine cells (about 35%) and primarily epithelial cells (about 57%). In contrast to ICCs, NPIs present the following important attributes: β cell expansion, significant ability for differentiation of epithelial cells into β cells, and comparatively higher responsiveness to glucose (43–45). Ironically, the use of NPI, however, was questioned by the high expression of xeno-antigens including sialic acid antigens, Hanganutziu–Deicher antigens, and Galα1–3Galβ1–4GlcNAc-R (21). Furthermore, just 50,000 NPIs per neonatal pancreas are not sufficient for its large-scale clinical applicability, and as a result, four neonate pig donors are required to treat a diabetic primate weighing 6–8 kg (46). However, less harvesting time and reduction in the housing cost of pigs led to the use of these islets in various trials (discussed later in this article). On the contrary, approximately 255,000 adult pig islets (APIs) with high purity (80–95%) can be isolated from an adult pig donor, and based on the Edmonton protocol, 5,000–10,000 IEQ/kg is needed to treat hyperglycemia (11, 32). Therefore, APIs are considered as a better source to correct hyperglycemia compared with ICCs and NPIs. Anticipating the efficient action of immune suppression agents as used in the Edmonton protocol, pig islets were transplanted in monkeys, and in **Table 1**, the success stories of independent clinical studies towards the restoration of glucose control upon pig islet transplantation are shown. Interestingly, both NPIs and APIs have been demonstrated to attain long-term survival of the islet graft in monkeys (**Table 1**).

**Table 1:** Various immune suppression regimens used to increase islet survival.

Immunosuppression therapy	Donor islets	Recipient nonhuman primates	MST	Reference(s)
Rapamycin + FTY720 + basiliximab + anti-CD154	API	Cynomolgus monkey	>187 days	(11)
CTLA4-Ig + rapamycin + basiliximab + anti-CD154	NPI	Rhesus monkey	>260 days	(12)
anti-IL-2R + anti-CD154 + belatacept + sirolimus (rapamycin)	NPI	Rhesus monkey	>187 days	(47)
CTLA4-Ig + rapamycin + anti-IL-2R + anti-CD40	NPI	Rhesus monkey	>203 days	(48)
MMF + CTLA4-Ig + LFA-3-Ig + anti-IL-2R + anti-LFA-1	NPI	Rhesus monkey	114 days	(49)
ATG + CVF + rapamycin + anti-TNF + anti-CD154 (+Treg)	NPI	Rhesus	>603 days	(50)

Rapamycin (mTOR inhibitor): FTY720 (targets the sphingosine-1-phosphate receptor 1), S1PR1 (an essential component for egress of lymphocytes from lymph organs into circulation), basiliximab (anti IL-2R inhibitor), anti-CD154 (anti-human CD154 monoclonal antibody ABI793 inhibits the interaction of CD40 receptors on antigen-presenting cells and CD154 on T cells; blocking co-stimulatory signals), anti CD40 (chimeric antibody: mouse Fab and rhesus IgG4 Fc fragments; blocks CD40 and CD154 co-stimulatory signals), MMF (mycophenolate mofetil; inhibits inosine monophosphate dehydrogenase), IMPDH (an essential enzyme for the de novo synthesis of guanine and adenine MMF and thereby prevents the proliferation of both T cells and B cells and also antibody production), LFA-3-Ig (alefacept; bindings at CD2 receptors of T cells and blocks proliferation and cytokine release), CVF (suppresses the deposition of circulating complement in an efficient manner to prevent hyperacute rejection), anti-TNF (etanercep).

T regs, regulatory T cells; API, adult porcine islets; NPI, neonatal porcine islets; MST, maximum survival time.

### 3.2 Instant blood-mediated inflammatory reaction in API and NPI and its therapeutic interventions

Apparently, in allo-transplantation of human islets, instant blood-mediated inflammatory reaction (IBMIR) results in the loss of a significant portion of transplanted islets within the first 60 min of intraportal transplantation (8). Initially, it was attempted to be overcome by increasing the number of transplanted islets in order to withstand an adequate islet mass for the post-IBMIR reversal of hyperglycemia (8). Exposure of cells and their extracellular matrix to a non-physiologic state—which is the intraportal bloodstream for the islets—might result in platelet consumption, activation of complement, and spikes of insulin release due to β cell death. The induction of tissue factor (TF)—constitutively expressed on islets as a result of inflammatory response on vessel endothelium, platelets, and neutrophils—has also been strongly suggested as pivotal for IBMIR (51, 52). Similar to the allotransplantation of islets, IBMIR-mediated loss has also been observed in pig-to-monkey intraportal islet transplantation studies (53, 54). Therefore, before testing these pig islets for human trials, it would be better if we could make the islets IBMIR-resistant (54). Xenogeneic IBMIR is also characterized by the activation of the complement and coagulation pathways (55, 56).

## 4 Injection of complement and coagulation inhibitors during transplantation

Various efforts towards overcoming IBMIR-induced islet damage have been made. Olle Korsgren’s group developed an *ex vivo* system that would mimic the post-transplantation situation (55). In this system, islets obtained from adult and fetal porcine pancreas were exposed to human blood for 5–60 min in heparinized polyvinyl chloride tubes. The addition of soluble complement receptor 1 (sCR1) along with heparin to these tubes prevented complement activation and also clotting by reducing the generation of coagulation factors: FXIIa-AT, FXIa-AT, and TAT (55). Furthermore, they tested the effect of sCR1 and heparin in pig to cynomolgus monkey (CM) transplantation studies. An abrupt increase in C-peptide, a marker of islet death, was found to be significantly reduced in CMs pretreated with sCR1 and heparin (56). van der Windt et al. examined pig or human islets that were selectively exposed to autologous, allogeneic, or xenogeneic blood with the following treatment conditions (1): low molecular weight dextran sulfate, (LMW-DS) for the prevention of blood clotting (2), nacystelyn (NAC) as tissue factor inhibitor (3), inhibitory protein for complement activation (hCD46) expressed on pig islets (4), compstatin (complement inhibitory drug), and (5) neutralizing anti-IgM antibody. All these treatments were shown to affect islet viability or function and C-peptide release. While LMW-DS, NAC, and hCD46 were not fully sufficient to block coagulation and complement activation and also the release of C-peptide by pig islets exposed to human blood, compstatin and neutralizing anti-IgM antibody treatment of human blood attenuated complement activation and prevented islet damage (57). This group also inhibited IBMIR by transplanting the pig islets in the gastric submucosal space of a pig in an allotransplantation model, wherein direct contact of blood and islets is avoided (58).

## 5 Genetic modification of pigs to avoid IBMIR and hyper-acute rejection

The IBMIR induced by porcine islets in human blood is most likely very similar to the allogeneic IBMIR, as it encompasses a gamut of innate immune components: complement proteins, macrophages, neutrophils, and platelet aggregation, which facilitate the destruction of porcine islet graft. To become an alternative strategy for a clinical islet replacement, pig islets must survive the imitation immune destruction dubbed as IBMIR to foster the long-term reversal of hyperglycemia (59, 60). Compared with API, neonatal islet cell clusters (NICCs) are more susceptible to bind anti-αGal antibodies due to the high expression of xenoantigen–galactose-α1,3-galactose (αGal). This condition is more likely to result in the application of complement activation, IBMIR, and thrombosis. Even though adult pig islet may survive IBMIR, it is also reported to undergo hyper-acute rejection in preclinical studies (61). Typically, as soon as pig tissues loaded with Galα (1, 3) are recognized by a nonhuman primate’s immune system, antibody ligation activates the classical complement pathway, leading to the formation of the membrane attack complex and culminating with cell lysis (60, 62). A significantly reduced amount of Gal epitopes on API compared with neonatal and fetal pig ICCs makes API less potent to activate anti-Gal-mediated IBMIR compared with its neonatal counterpart. Thus, removal of the αGal epitope from the islet surface is a reasonable option, as it may add one more safety feature to the islets used in transplantation.

Interestingly, Rita Bottino’s group exposed αGal knockout (GTKO) pig tissues and islets separately with human serum *in vitro*. The presence of IgG and IgM on the islet surface suggested the presence of non-Gal antigens (58). N-glycolylneuraminic acid, encoded by cytidine monophospho-N-acetylneuraminic acid hydroxylase (CMAH), and Sid antigen, encoded by β1,4 N-acetylgalactosaminyltransferase, are two noticeable non-Gal antigens identified on pig cells in addition to αGal (63, 64). In this pursuit, Tector’s group demonstrated that the removal of both CMAH and GGTA1 gene is necessary to inhibit xeno-immune reactions against pig organs in a xenotransplantation setting (65, 66). This group also explored the possible role of β4GalNT2 gene in xeno-rejection, but the role of β4GalNT2 in pig islet xenotransplantation is still unexplored (67, 68).

Additionally, transgenic expression human CD46 (blocks C3 convertase complex), CD55 (regulates cell susceptibility to complement attack by inhibiting the formation of C3 and C5 convertases), and CD59 (regulates the final stage of the complement enzyme cascade by blocking C8 and C9 and the polymerization of C9) are still needed to further inhibit the activation of complement and thrombosis in the intraportal vein upon pig islet transplantation (69). Furthermore, the transgenic expression of coagulation system inhibitors, *viz*., human tissue factor pathway inhibitor (hTFPI) and CD39, providing anti-thrombotic and anti-inflammatory effects, is beneficial to the islets (69, 70). In another study, the transgenic expression of hemo oxygenase-1 rendered protection to the islets from ischemic injury during isolation until engraftment and vascularization after liver transplantation (71). **Table 2** underscores the positive effects of the aforementioned genetic modifications along with an immune suppression regimen on increasing the maximum survival time of pig islet grafts in nonhuman primates.

**Table 2:** Exploring genetic modification in donor pig islets with immune suppression regimen to increase the post-transplantation survival time.

Type of genetic modification	Donor islets	Recipient nonhuman primates	Immune suppression regimen	MST(days)	Reference(s)
**GTKO**	API	Rhesus monkey	MMF + anti-CD154mAb + anti-LFA-lmAb + CTLA4-Ig	249	(48)
**hCD46**	API	Cynomolgus monkey	MMF + ATG + anti-CD154mAb	>396	(72)
**hCD55**	Fetal	Cynomolgus monkey	Cyclosporine + steroids + cyclophosphamide or brequinar	7	(73)
**GTKO/hCD55/hCD59**	NP!	Baboon	MMF + ATG + tacrolimus	28	(74)
**Multi-transgenic** **(GTKO/CD46 universal expression, and beta cell**-**specific hTFPI/CD39/porcine CTLA4-Ig**	API	Cynomolgus monkey	MMF + ATG + anti-CD154mAb	5 months	(75)
**GnT-III**	API	Cynomolgus monkey	None	5	(76)

GTKO (galactosyltransferase gene knockout pig), hCD46 (membrane cofactor protein that controls complement activation), hCD55 (also known as hDAF; decay-accelerating factor that accelerates the decay of the C3 and C5 convertases), hCD59 (human complement regulatory protein; CRP, perturbs the complement activation cascade by inhibiting the formation of the membrane attack complex during the final stage of complement activation), hTFPI (human tissue factor pathway inhibitor; inhibits the activation of TF : FVIIa coagulation protease pathways, leading to fibrin deposition and activation of platelets during the course of hyperacute rejection), CD39 (via its ATPase activity, it decreases platelet activation and inhibits clotting), anti-LFA-1 (anti-lymphocyte function-associated antigen-1 monoclonal antibody; efficient in eliminating memory T cells).

ATG, antithymocyte globulin; CM, cynomolgus monkey; GnT-III, N-acetylglucosaminyltransferase III; MMF, mycophenolate mofetil; MTS, maximum survival time.

### 5.1 Islet encapsulation: Capable of evading IBMIR and gradual activation of islet-specific immune responses

The long-term survival of pig islets will only be possible if we could promote the survival of the transplanted islets from the deleterious effects of IBMIR and donor-specific immune responses during chronic rejection. Encapsulation of islets using a semipermeable membrane is one of the strategies to protect the islets in such way that it could allow normal insulin secretion in response to fluctuating blood glucose levels and permits the diffusion of oxygen and essential nutrients. Based on the size of the capsule and the number of islets that they can accommodate, three encapsulation systems have emerged: macro-encapsulation (size in centimeters), micro-encapsulation (250–1,000 μm), and nano-encapsulation (<100 μm). In macro-encapsulation, first proposed by Algire, Prehn, and Weaver in the 1950s, a large number of pancreatic cells entrapped in a capsule of centimeter range were tested (77). Furthermore, the oxygenated islet macrocapsules generated by Ludwig et al. demonstrated the long-term survival of human allogenic islets without any immune suppression (78). Similarly, one case report published by Elliot et al. claimed the very long-term survival of macroencapsulated pig islets (9.5 years) without immune suppression (79). βair^®^ devise, TheraCyte™, VC-01, and cell pouch and islet sheet devices are examples of the macro-encapsulation of islets that are currently under preclinical studies. All these devices are transplanted at subcutaneous sites without immune suppression (80–86) (**Table 3**).

**Table 3:** Enumeration of devices containing macroencapsulated islets being used in various preclinical and clinical trials.

Name of device	Pre-clinical/clinical trials	Design advantage	Reference(s)
β-Air	Transplantation of porcine islets into monkeys Clinical trial with human islets transplanted into humans	Promises adequate oxygen supply and comparatively improved protection from recipient immune system	(80, 81)
3D printed vascularized device	Transplantation of human islets in mice	Contains growth factors for optimized vascularization of islets	(82)
Bioplotted scaffold	Transplantation of human islets into mice without immunosuppression	Enhanced vascularization and efficient diffusion owing to adjustable pore size; also avoids islet clumping	(83)
Silicon nanopore membrane device	Mouse islets into pig islets	Adequate pore size (10 nm) and more cell viability	(84)
TheraCyte	Lewis rats were islet donors, and alloimmunized, diabetic Wistar–Furth rats were used as recipients	More vascularization and optimum inner membrane pore size (0.4 μm) to embargo immune response	(85)
Encaptra	Implantation of pancreatic progenitor cells into mice. Clinical trial: implantation of pancreatic progenitor cells into humans	The device allows the growth of pancreatic progenitor cells	(86)

Subsequent to their success in NHP models, micro-encapsulated pig islets are being used to correct hyperglycemia in T1D patients and have shown promising results in countries including the United States (**Table 4**). These pilot clinical trials have confirmed the biosafety of islet-encapsulated capsules, but the efficacy of the treatment is still under review. The immunological responses against microcapsules pose a difficulty in choosing the right microcapsule material without having the potential threat of imminent inflammatory immune responses (93). Therefore, to avoid an adaptive immune response to microcapsule materials, several immunosuppressive coating molecules, *e*.*g*., Fas ligand (FasL), tumor necrosis factor (TNF)-related apoptosis-inducing ligand, and CD200, have been immobilized on the surface of these islet-containing capsules (94–96). Above all, FasL has been the most extensively studied strategy to eliminate graft-specific T effector cells. Yolcu et al. recently demonstrated the long-term engraftment of allogeneic and xenogeneic islets coated with FasL protein combined with short-term rapamycin treatment (97). Additionally, micro-encapsulation of islets with hydrogel containing immobilized inhibitory peptide for interleukin-1 receptor efficiently protected the islets from IL-1β, TNF, and interferon-γ *in vitro* and also *in vivo* by β cell-specific T cells (98–101). Furthermore, immobilization of CXCL12 and CCL22 has proven to be very effective in different studies in prolonging the protection of islet allograft and restoring normoglycemia without immune suppression (102).

**Table 4:** Devices containing microencapsulated islets used in various preclinical and clinical trials.

Islet type	Pre-clinical/clinical trials	MST	Reference(s)
API	Microencapsulated in alginate and transplanted in the kidney capsule of CM	6 months	(87)
Human islets	Type 1 diabetes patients received an intraperitoneal transplant of microencapsulated human islets	35 months	(88)
API	Microencapsulated in single alginate coats and double alginate coats, separately, and transplanted into diabetic B6AF1 mice	>6 months	(89)
NPI	Alginate microencapsulated islets in CM	36 weeks	(90)
NPI	Alginate microencapsulated islets in NOD mice	8 weeks	(91)
API	Alginate–polylysine–alginate microencapsulated islets intraperitoneally in CM	3 months	(92)

API, adult porcine islets; NPI, neonatal porcine islets; CM, cynomolgus monkey; MST, maximum survival time.

## 6 Stem cells as the next alternative source to generate α and β cells

Typically, the cell replacement therapies targeting T1D use a two-pronged approach (1): replenishment/restoration of β cell mass by transplantation and (2) rescuing β cell mass by inducing immune suppression. As discussed above, the allo- and xenotransplantation of islets made great promises to manage overt hyperglycemia, but destruction of graft after tapering off immune suppression more often results in chronic rejection. To this end, researchers are now seeking answers in the regeneration of β cells from stem cells and their transplantation in the recipients. Recently, stem cell biologists systematically recapitulated the needed differentiation events to develop beta cells during pancreas development, and they have pursued the conversion of embryonic stem cells in beta-like cells in a dish. At the time of transplantation, these *in vitro*-transdifferentiated cells were not functionally mature but were demonstrated to express both insulin and glucagon (103–108). Though not functionally mature, these cells were converted into mature beta cells on transplantation in mice and surprisingly maintained normal glucose levels after ablation of the pancreas (106). Following these pioneering studies, Rezania et al. and Paliguca et al. made insulin-producing functional beta cells after slightly modifying the array of reagents (23, 24). This process further needed to be scaled up as about half to a billion cells per transplant, which is equivalent to 10,000 IEQ/kg (8), are needed to restore hyperglycemia in human.

### 6.1 Strategy to generate beta cells from iPSCs: Game-changer strategy

Though the concept of employing iPSCs was initially very attractive in T1D, scaling this strategy up involved the activation of four Yamanaka transcription factors (*Oct-3/4*, *Sox-2*, *Klf-4*, and *c-Myc*) (109), thus posing several issues. Yamanaka’s group orchestrated the reprogramming of human fibroblasts through the retroviral transduction of the aforementioned transcription factors (109). This approach was questioned by the tumorigenic potential of retroviruses and the activation of oncogene c-Myc during the process. Therefore, to avoid imminent disapproval by regulatory agencies, the RNA-based Sendai virus (SeV)-centered approach was developed. SeV-mediated reprogramming was proven to be efficient and highly reliable as SeV replicates outside of the cell, thereby preventing integration into the host DNA (110).

More recently, the conversion of functional beta cells was reported from iPSCs by adding multiple chemical inducers and inhibitors of Wnt and Notch pathways in a particular sequence. Based on the sequential activation of genes, this mimics the ontogeny of the pancreas development over 30 days using 2D and 3D culture techniques. Importantly, the crux of this process is the conversion to mono-hormonal, *i.e*., insulin-positive, cells in the end (23, 24). This seven-stage process to develop functional beta cells involves the use of various inhibitors at each stage: vitamin C until stage 4, a combination of ALK5iII inhibitor and thyroid hormone (T3) from stages 4 to 7, GSKixx at stages 5 and 6, and N-Cys at stages 6–7 turned out to be the key components. Rezania et al. outstandingly delineated the roles of each compound in terms of the stage-specific signatures using RT-qPCR (24). These iPSC-derived beta cells were then transplanted under the kidney capsules of immunocompromised mice, and human insulin was detected (23, 24).

Douglas A. Melton’s group compared the insulin secretion of iPSC-derived β cells with human islets. The amount of insulin secreted by primary human beta cells and iPSC-derived β cells was found to be 1.6 ± 0.2 and 3.6 ± 0.7 μIU/10^3^, respectively. They have also measured the total insulin content per cell, which was comparable: 240 ± 50 μIU/10^3^ cell (SC-β clusters) and 200 ± 40 μIU/10^3^ (primary islets). Furthermore, a similar insulin ratio and Ca^2+^ (upon glucose challenge) both in primary human islet cells and SC-β cells supported the SC-β cells as an imminent alternative sanctuary of insulin-producing cells to reverse hyperglycemia.

### 6.2 Encapsulation of beta cells and reverting hyperglycemia

Transplantation of β cells derived from T1D patient-derived iPSCs can correct hyperglycemia. However, these SC-β cells succumb to destruction due to β cell-specific autoimmunity in these individuals (111). The use of a systemic immunosuppressant is therefore needed to protect these cells from autoimmune attack. To avoid lifelong immune suppression, encapsulation is one of the most plausible solutions. Daniel Anderson and Song’s group independently attempted the encapsulation of SC-β cells and tested it in immunocompetent mice. Anderson’s group recently used triazole–thiomorpholine dioxide (TMTD) alginate for encapsulation, showing that it could inhibit fibrosis in both rodents and nonhuman primates (112). Interestingly, TMTD alginate-encapsulated SC-β cells provided long-term glycemic correction without immunosuppressive therapy in immune-competent diabetic C57BL/6J mice for 174 days. However, naked SC-β cells were unable to correct overt hyperglycemia irrespective of the site. Song et al. encapsulated the islets and stem cell-derived β cells in polylactic acid-derived microporous 3D printed device and studied the outcomes when transplanted in mice. Insulin secretion was successfully reported for 12 weeks. However, the group did not evaluate the extent of immune tolerance to this material (112). Like porcine islet tx, iPSC-derived beta cell therapy for patients with T1D has a few limitations. The key barrier of transplantation is the control of the immune response. However, the autologous transplantation of iPSCs might be useful for avoiding rejection because they are not thought to initiate immune responses (113, 114). The second disadvantage is that there is a potential for the transplanted cells to become cancerous. The c-Myc gene, one of the Yamanaka factors, is known to cause cancer in iPSCs (115), but oncogenesis can be prevented by using L-Myc instead of the c-Myc gene (116). The third limitation is the possibility that the insulin secretion of the transplanted cells may be insufficient. iPSCs-derived islet cells transplanted into mice reportedly secreted insulin for several months or more after transplantation (PMID: 30623004). However, long-term studies are required to investigate whether the insulin secretion capacity is sufficient for clinical application.

## 7 Future perspectives

Xenotransplantation of pig islets has proven equally effective as human islets in preclinical trials; therefore, scientists are striving hard to make this approach a clinical reality. The idea of pig islet transplantation may overcome the shortage of good-quality islets. Strong immune suppression with xeno-antigens and IBMIR is a major challenge faced by pig islets’ transplantation in preclinical studies. Extensive gene editing by user-friendly CRISPR–Cas9 system promises to rewrite the bright future of pig islet transplantation by developing IBMIR-resistant islets. Though ideal biocompatible material is still a matter of debate, encapsulation techniques offer overall protection of islets from the recipient’s immune system. Several preclinical trials of encapsulated pig islets are underway with some promising results. Excitingly, 9.5-year survival of encapsulated neonatal pig islets in a human trial in Auckland has been reported (76). Meanwhile, the groundbreaking work of Alireza Rezania, DA Anderson, and D Melton have leveraged the use of stage 7 (functional) beta cells to cure T1D. Contrary to pig islets, encapsulated SC-derived β cells equivalent to 10,000 IEq/kg of T1D patients might overt T1D with the minimal use of immunosuppressant if derived from iPSCs obtained from the same T1D patient (**Table 5**).

**Table 5:** Comparison between pig islets and stem cell (SC)-derived β cells for the imminent cure of type 1 diabetes.

	Pig islets	SC-derived β cells
**Advantages**	Promise to overcome donor shortage Unlimited supply Encapsulation to render long-term function Sufficient insulin secretion capacity for long-term survival in mouse, monkey, and human	Promise to overcome donor shortage Encapsulation to render long-term function Sufficient insulin secretion capacity for long-term survival in mice
**Disadvantages**	Immunosuppression a major challenge High risk of rejection PERV infection	Immunosuppression a major challenge, as these are never tested prevalent immunosuppressant (Edmonton’s protocol) Scaling up: challenging—only a promise Not tested in large animals (*viz* nonhuman primates)

In conclusion, SC beta cells for the treatment of T1D increase quality-adjusted life years (115, 117) and prevent complications, although a reduction in manufacturing costs will be essential to achieve cost-effectiveness. By scaling up the manufacturing, as promised, facilitating the supply chain management, and reducing the manufacturing costs, iPSC-based β cell replacement therapies may become a tangible reality.

## Author contributions

Conceptualization and writing: RN, AS, and AN, review and editing: MP, AB, and BL. RN and AS: instrumental in revising the manuscript. All authors contributed to the article and approved the submitted version.
